# GNN-ML-FRL: a graph-enhanced meta-adaptive federated learning framework for scalable pest identification and ernvironmental modeling

**DOI:** 10.1038/s41598-026-49344-y

**Published:** 2026-05-01

**Authors:** Mahalakshmi, Suma T, Sandeep Kumar Mathivanan, Sangeetha R, Rose Bindu Joseph, Sangeetha S.K.B

**Affiliations:** 1 Department of Mathematics, Nitte Meenakshi Institute of Technology, Nitte (Deemed to be University), Bengaluru, Karnataka India; 2 Department of Mathematics, New Horizon College of Engineering, Bengaluru, Karnataka India; 3 Department of Computer Science & Engineering- AI & ML, KG Reddy College of Engineering and Technology, Hyderabad, Telangana, 501504 India; 4 Department of Computer Science and Engineering,School of Computing, Vel Tech Rangarajan Dr. Sagunthala R&D Institute of Science and Technology, Avadi, Chennai, 600062 India; 5 Department of Mathematics, Dayananda Sagar College of Engineering, Bangalore, India; 6 Manipal Institute of Technology Bengaluru, Manipal Academy of Higher Education, Manipal, India

**Keywords:** Precision agriculture, Graph neural networks, Federated learning, Meta-learning, Pest recognition, Genotype-informed modeling, Computational biology and bioinformatics, Ecology, Ecology, Environmental sciences, Mathematics and computing

## Abstract

Heterogeneous agro-ecological factors, insect breeding, and climate change are serious challenges to sustainable agricultural management. The study proposes a graph-enhanced meta-adaptive federated learning framework (GNN-ML-FRL) to address the challenges in precision agriculture. The proposed framework integrates Federated Learning (FL) for collaborative training of models in a decentralized manner across geographically distributed farms, Meta-Learning (ML) for rapid adaptation to changing environmental factors, and Graph Neural Networks (GNNs) for capturing spatial dependencies among agricultural entities. A comprehensive multivariate IoT environmental dataset with 52.56 million time-series observations gathered from 500 dispersed sensors over a 12-month period, the IP102 insect pest recognition benchmark (75,222 images across 102 species), and curated genomic datasets from MaizeGDB and the Rice Annotation Project Database for genotype-informed modeling are the three standardized datasets used to assess the framework. Experimental results show statistically significant improvements (*p* < 0.01) over CNN and graph-based baselines, achieving 89.3% Top-1 accuracy, 7.8% higher generalization performance, and 12.4% reduction in prediction loss across geographically unseen farms. SHAP-based explainability further indicate that environmental accuracy-related features contributed nearly 63% positive influence, while loss-related factors contributed 37% negative influence, validating model robustness. Geographic generality is confirmed by site-out validation using IoT data, and resilience is improved under varied crop conditions by genotype-informed graph modeling. The findings show that a scalable and statistically sound framework for data-driven pest identification and environmental modeling in precision agriculture may be achieved by combining spatial graph reasoning, meta-adaptive learning, and decentralized training.

## Introduction

The global economy relies on agriculture, but the sector is being subjected to pressure to meet the demands of an expanding global population in terms of food security. Pests, scarce resources, and climate change are significant challenges with conventional methods of farming. Precision Agriculture (PA) is the management of the agricultural production process by means of information technology and multiple sources of information. It utilizes Artificial Intelligence (AI), sensor systems, and data analysis to maximize sustainability, reduce resource consumption, and maximize production. The current methods have limitations which complicate the application.

The agricultural ecosystem is the key to precision farming. Precision farming is a complicated task to predict and regulate agricultural activities due to the high diversity of ecosystems that involve various types of soil, climatic factors, and crops. Many of the conventional ways of resource management, irrigation, and pest management are not capable of making very accurate decisions or change-reacting in real-time. Agriculture’s temporal and spatial relationships are challenging to be depicted by utilizing existing methods, which are primarily based on human expertise.

One of the important factors in precision agriculture involves the application of machine learning algorithms to parse massive data collected from sensors, satellite imagery, and UAVs (Unmanned Aerial Vehicles). In crop yield prediction of agricultural crops, disease detection, and the allocation of resources as efficiently as possible, supervised learning methods such as decision trees, Support Vector Machines (SVMs), and random forests are commonly used. The ability of these classic models to process unstructured data, e.g., recordings of real-time sensor readings that vary significantly with time, is limited. Classic models require vast sets of labeled data, which are time-consuming and costly to acquire, for rare or new events like insect infestations.

Agriculture has been enhanced by Deep Learning (DL) methods, when applied in tasks such as crop classification from high-resolution images and disease identification. Convolutional Neural Networks (CNN) models have significant limitations, even while CNNs have been widely applied for image-based crop evaluation. These include difficulty in embedding domain knowledge such as soil or weather, large training data requirements, and a failure to generalize to a multitude of environmental conditions. CNNs can be applied to clearly defined tasks but cannot capture the multivariate, multilevel, complicated relationship between real-world agricultural systems, such as the interaction between soil health, pest infestations, and climate change.

Central data processing in existing precision agriculture systems is another challenge. Classic approaches need all the data of various farms or plots of land to be collected, processed, and interpreted at one server, causing bandwidth, security, and data privacy concerns. This central approach does not have the scalability to carry out on large scales over various agricultural zones, particularly in developing regions with low connectivity. Recent research has begun exploring advanced AI approaches such as Graph Neural Networks (GNNs), Reinforcement Learning (RL), and Federated Learning (FL). GNNs have proven to be promising on problems ranging from social network analysis to protein interaction prediction due to their ability to outperform traditional methods in encoding spatial dependencies in graph-structured information.

The complex interactions among variable environmental states, crops, pests, and resources across multiple geographical scales can be handled in agriculture using GNNs. GNNs are robust in understanding agricultural systems since they incorporate the diverse site-to-site interactions within a field, farm, or region, in contrast to CNNs, which are designed for grid-type data. RL has proven potential in optimizing decision-making processes. By interacting with the environment and learning through reward or penalty feedback, the RL agents can acquire the optimum practices for processes such as fertilizer management, irrigation scheduling, and insect control. In comparison with traditional rule-based systems or even supervised machine learning models, the ability to learn from experience and real-time adaptability has enormous benefits. The robustness and scalability are also enhanced by continuous learning and adaptation to changing conditions made possible by the union of RL with real-time data streams. Another potential solution to resolve decentralized data processing and data privacy issues is FL. Without the sharing of raw data, FL enables different parties, different farms or sets of farms, to collaborate in building machine learning models. Sensitive data are not disclosed because both parties train a local model on respective data and transfer model updates only. This approach is appropriate for large-scale, real-time agriculture environments because it not only enhances data privacy but also reduces high-volume data transfer demands.

There are several challenges that must be overcome before these techniques can be used in actual agricultural systems. Although techniques such as GNNs and RL are learning-based, it requires extensive adaptation for a specific domain to consider local variations in crops, soil, and climate. With use in new areas or untapped farming problems, such as infestations of unusual insects or strange weather patterns, the performance of the system may degrade even using cutting-edge methods. Also, it remains challenging to integrate data from various sources, i.e., environmental sensors, UAVs, and satellite imagery, into one AI system.

To prepare the data for machine learning algorithms, it must be preprocessed since it is noisy, unsystematic, or incomplete. A source of complexity arises from the necessity of coordinating data from numerous sources, especially in big agriculture enterprises. Another challenge is the operational complexity of real farm systems. The proposed AI models are advanced and capable of operating autonomously in complex field environments with minimal or no intervention by humans. To real-time decision-making that responds to shifting environmental conditions such as temperature gradients, soil water content, or pest cycles, this involves open interfacing with current agriculture practices and systems.

Graph Neural Networks, Federated Learning, Meta-Learning, and Reinforcement Learning are controlled by a unified optimization scheme in the proposed framework of GNN-ML-FRL, which is not only innovative in its ability to integrate the most recent AI methodologies but also in its ability to integrate a unified learning paradigm. The proposed method integrates joint spatial modeling, collaborative learning, rapid domain adaptation, and adaptive decision learning in a single framework, as opposed to the earlier work that has applied these methodologies in a disjoint and sequential manner.

Meta-Learning enables agro-ecological diversity, Federated Learning preserves locality in collaborative learning, Reinforcement Learning optimizes decisions in a dynamic environment, and Graph Neural Networks encode spatial dependencies among farms. Thus, the methodological contribution of the proposed work is the unified meta-adaptive federated graph learning method that enables scalable, local, and dynamic precision agricultural modeling.

**Key** **Contributions** **and** **Novelty** **of** **the** **Proposed** **Framework**

Beyond current hybrid AI models in precision agriculture, the suggested GNN-ML-FRL framework provides several unique additions. First, the suggested framework incorporates Graph Neural Networks, Federated Learning, or Meta-Learning into a single optimization architecture, allowing for joint learning of spatial dependencies, cross-domain adaptation, and decentralized collaboration, in contrast to previous works that use these paradigms separately or in loosely coupled pipelines. Second, the use of meta-learning enables quick adaptability to a variety of agro-ecological circumstances with little retraining, a feature that is uncommon in federated agricultural models now in use.

Thirdly, as opposed to the static prediction approaches used in previous studies, by integrating reinforcement learning, there is dynamic choice optimization through real-time feedback from the environment. Fourthly, there is multimodal data fusion, where genetic, environmental, and vision data are used to increase robustness in generalization and prediction. Lastly, the suggested architecture can sustain good performance even in dispersed farms while at the same time promoting scalability and privacy through federated optimization. All these contributions are for a next-generation precision agriculture system that is cohesive, flexible, and scalable.

The main contributions are.

- The study proposes hybrid CNN-GNN architecture for large-scale insect pest recognition, demonstrating statistically significant improvements over established baselines on the IP102 benchmark.
- The study introduces a graph-based encoding of SNP-derived principal components for crop accessions, enabling genotype-informed predictive modeling using curated releases of MaizeGDB and RAP-DB.
- The study incorporates meta-learning strategies to enhance adaptation across pest species, environmental zones, and crop accessions.

The main objectives are.

1. To design a scalable graph-based learning framework that integrates Graph Neural Networks and Federated Learning for decentralized agricultural data modeling, enabling spatial reasoning across farms while preserving data locality.
2. To develop a multimodal learning architecture combining image-based pest recognition, environmental IoT time-series modeling, and genotype-informed graph representations to improve predictive robustness across heterogeneous agro-ecological conditions.
3. To incorporate meta-learning mechanisms that enhance cross-domain adaptability, allowing the system to generalize across unseen farms, climatic zones, and crop accessions with minimal retraining.

## Related study

There is a need for agriculture to address challenges of sustainability and climate change without undermining its food production potential with smaller resources. Precision Agriculture (PA) is the mainstream strategy in addressing these challenges, replacing conventional forms of agriculture. Precision agriculture utilizes new technology in the form of sensors, satellite imaging, UAVs, and Machine Learning (ML) for enhanced farm output, minimized wastage, and maximized resource utilization. One of the challenges of classic PA approaches is the centralized processing of data. It is necessary to forward data collected from multiple sources, satellites, UAVs, and remote sensors into a common server for processing. Other than creating privacy issues for information, this collection heightens bandwidth to enable real-time decision-making^1^. Such edge-facilitated decentralized systems that are capable of handling data processing at the edge, provide privacy, and have good prediction and recommendation performance are becoming more important^2^.

Machine Learning (ML) techniques have recently been applied in PA to address pest control, yield forecasting, and disease detection. A few farm applications have demonstrated the effectiveness of traditional machine learning methods like Support Vector Machines (SVMs), random forests, and decision trees. The complexity and variability of farmland pose problems for these models^3^. There are challenges to model generalization since weather, soil humidity, pest infestation, and crop growth patterns are dynamic. Supervised algorithms also need huge amounts of labeled data, which is not feasible for the infrequent cases of pest infestation or extreme weather patterns^4^.

Deep Learning (DL) technology in the form of Convolutional Neural Networks (CNNs) are capable of analyzing high-resolution images and making predictions related to crop health and disease diagnosis^5^. CNNs are not equipped to deal with the complex spatiotemporal interactions between the factors influencing the health of crops despite their benefits. It is constrained by the grid basis of static images, which is presently insufficient to depict the spatial relations within blocks in farms^6^.

Combining heterogeneous sources of data such as sensor observations, satellite images, and meteorological prediction into homogenous models with the ability to decide are another future challenge in precision farming. It is challenging to combine data sources well for accurate prediction since data are of different format, size, or time dimension^7^. By depicting farming plots as graphs with nodes as spatial coordinates (e.g., pieces of land) and edges as interactions between them (e.g., weather conditions such as temperature or humidity levels), graph-based approaches have proved applicable here. These geospatial interactions are described by GNNs which allow the system to learn interdependencies of several variables and improve the quality of its predictions^8^.

Precision agriculture, data centralization and privacy issues have been addressed through the implementation of FL, a distributed learning technology. Federated learning enables many farms or other agricultural interest groups to jointly train machine learning models without disclosing local data. By sharing model updates, data privacy is preserved, and communication overhead is minimized. This method is relevant to the agricultural industry, where several stakeholders are not willing to share confidential information due to competitive or privacy issues^9^. FL has already proved to be promising in almost all other fields as well, ranging from medicine and money to increasingly being studied for application in agriculture in order to facilitate data-efficient collaborative learning without compromising privacy^10^.

Another innovation in the creation of adaptive and autonomous systems is RL for precision agriculture. Smart agents that learn the best action through trial and error in varying environments can be created as a spin-off of reinforcement learning. Utilizing environmental feedback in real-time, RL has uses in agriculture for optimal crop protection methods, irrigation planning, pest management, and resource allocation. In contrast to conventional rule-based systems that do not explicitly reveal the depth of real-world agricultural systems, RL can learn and adapt based on experience under changing conditions. A number of studies have shown how RL is implementable in agricultural decision-making by encouraging findings in areas of insect pest control and irrigation scheduling^11,12^.

Some of the important challenges can be overcome by increased usage of AI methods like deep learning, federated learning, and reinforcement learning in precision agriculture. These are safeguarding data privacy, supporting real-time decision-making, and improving model accuracy^13^. Challenges also exist but particularly concerning real-time deployment of these systems over diverse agricultural settings. Such applications require highly generalized models with the ability to generalize over a wide range of environments based on soil variation, weather, and pest regimes. Integration of several AI techniques into a single system to perform various tasks, for instance, forecasting yield, disease prediction, and pest identification, is another significant challenge. It would involve development of hybrid AI models that leverage the strengths of multiple approaches^14^.

Although astronomical advancements in agri-tech with the help of AI, there are implementational realities such as cost of adoption, infrastructure deficiency in rural areas, and training of farmers that severely complex adoption. For AI systems to collect, process, and analyze data in agriculture, there needs to be a robust infrastructure, which is not possible in rural areas^15^. The scalable, affordable, and user-friendly solutions are implemented so that access to AI in agriculture is made available to individuals^16^.

The use of AI technology is being driven by the transition towards environmentally sustainable farming practices. Precision agriculture reduces environmental complexities, increases production, and conserves resources than the traditional farming practices. Artificial intelligence technologies allow us to use inputs like water, fertilizers, and pesticides in a precise manner, reducing the total usage and reducing the negative environmental effects^17^. AI can make monitoring of biodiversity and soil condition easier, both of which are two of the key components of sustainable agriculture. AI can increase global access to food security with reduced environmental footprint of agriculture by making farming practices sustainable^18^.

By maximizing utilization of resources, enhanced decision-making, and sustainability, the integration of advanced AI techniques like graph neural networks, federated learning, and reinforcement learning into precision agriculture performs better in farm production. A number of challenges exist in affecting model generalization, data integration, and real-time deployment of AI systems. AI can transform agriculture and achieve the potential of augmented productive and sustainable farming activities using these innovations^19,20^.

From Table 1, the studies find the opportunities and challenges in agricultural technology. While traditional methods are better with formal data, standard machine learning algorithms are not effective in processing high-dimensional, unstructured data like satellite imagery and sensor reading data that are typical to agriculture. CNNs require large amounts of labeled data, which may not always be readily available in farms. CNNs do not perform as well with sequence or time data, such as forecasting agricultural crops or disease detection based on past trends. Time-varying agricultural practices can be a challenge to GNNs but could potentially describe spatial relations, such as in pest and soil dynamics.

**Table 1:** Comparison Analysis.

Parameter	Traditional Machine Learning	Convolutional Neural Networks (CNNs)	Graph Neural Networks (GNNs)	Reinforcement Learning (RL)	Federated Learning (FL)	Meta- Learning	Deep Learning for Time Series	Synthetic Data Generation (SDG)	IoT and Sensor Data Integration	Climate- Smart Agriculture
Type of Data	Structured data (tabular, numerical)	Image data (satellite, drone imagery)	Graph-structured data (spatial relationships)	Time-series data, sequential decisions	Distributed data across multiple nodes	Few-shot learning tasks; small datasets	Temporal data (weather, pests, crop growth)	Synthetic data for rare events or training augmentation	Real-time sensor data (moisture, temperature, etc.)	Climate and environmental data
Primary Applications	Crop Yield Prediction, Pest Detection, Disease Classification^21^	Disease Detection, Weed Identification, Crop Health Monitoring^23,24^	Soil–Plant Interaction Models, Pest Prediction Models, Environmental Modeling^25^	Irrigation Control, Pest Management, Nutrient Allocation^27,28^	Collaborative Pest Management, Weather Prediction, Data Privacy^29,30^	Adaptive Pest Management, Dynamic Resource Allocation(^31,32^	Pest Outbreak Prediction, Crop Yield Forecasting, Weather Prediction^33,34^	Training Augmentation for Rare Events, Crop Prediction Models for Rare Events^35^	Crop Health Monitoring, Resource Management, Environmental Assessment^26,29^	Water Conservation, Sustainable Crop Selection, Climate Adaptation^22,23^
Model Complexity	Low to moderate	High (due to deep layers and large datasets)	Moderate to high (requires graph construction and edge learning)	High (requires interaction and feedback for learning)	Moderate to high (depends on distributed system setup)	High (requires training across multiple tasks)	High (requires large time-series datasets and complex architectures)	Moderate to high (depends on GAN/VAE architecture)	Moderate (requires real-time data processing and integration)	High (requires integration of multi-source environmental data)
Data Requirements	Labeled data for supervised learning	Large, labeled image datasets	Graph-structured data, labeled environmental data	Extensive time-series data for training	Local data storage at each node, model updates shared	Few-shot data sufficient for adaptation	Long-term historical data for weather and crop growth trends	Limited real-world data, synthetic data is generated for model robustness	Continuous data collection; real-time streaming	Multi-source environmental data with high variability
Strengths	Simple, interpretable models; works well with structured data	Best for image analysis, accurate crop disease identification	Excellent at capturing spatial dependencies in large fields	Autonomous decision-making, adapts to changing environments	Preserves data privacy, can aggregate knowledge from various farms	Rapid adaptation to new environments with minimal data	Excellent at forecasting time-dependent phenomena	Overcomes data scarcity for rare or underrepresented events	Enables continuous monitoring and real-time interventions	Tailored to specific regions’ climate conditions; adaptive solutions
Limitations	Struggles with high-dimensional, unstructured, or temporal data	Needs large amounts of labeled data; limited to image-based tasks	Computationally intensive; may struggle to generalize to diverse conditions	Requires extensive data for training; slow convergence in real-time applications	Requires high-bandwidth and robust infrastructure; slow model updates	Requires fine-tuning and complex implementation; limited in real-world applications	Requires long historical data; prone to overfitting if not regularized	Data quality can vary; needs careful validation of synthetic data	Sensor calibration and maintenance; synchronization issues	Complex integration; needs large-scale infrastructure
Relevance to Agricultural Challenges	Effective for structured data analysis like crop yield and pest prediction	Essential for real-time crop health monitoring using drones or satellites	Ideal for modeling soil-health interactions and pest propagation in large fields	Provides adaptive decision-making for irrigation, pest control, and fertilizer application	Enables decentralized data processing with privacy preservation; useful for multi-farm collaboration	Adapts rapidly to changes in environmental conditions, useful for dynamic farming scenarios	Effective in predicting crop growth and pest outbreaks based on temporal trends	Useful for increasing training dataset size, particularly for rare phenomena	Provides a comprehensive view of the farm with real-time updates; enhances field management	Assists in planning for climate-resilient farming practices by adjusting to environmental changes
Example Techniques	Decision Trees, Random Forests, SVM, k-NN	VGG16, ResNet, EfficientNet	Graph Convolution Networks (GCN), GraphSAGE, GAT	Deep Q-Learning, Proximal Policy Optimization (PPO)	Federated Averaging (FedAvg), Federated Stochastic Gradient Descent (FedSGD)	Model-Agnostic Meta-Learning (MAML), Reptile	Long Short-Term Memory (LSTM), Transformers, GRUs	Generative Adversarial Networks (GAN), Variational Autoencoders (VAE)	Edge computing with sensors, Cloud Integration for farm management	Integration of climate prediction models with AI algorithms

^35^Presents a cloud-based crop recommendation model, aiming to optimize agricultural practices through transformative insights for better crop management. ^36^Investigates the health impacts of synthetic agrochemicals, proposing a predictive framework using machine learning approaches to analyze their effects on human health. ^37^Highlights a cloud-IoT and UAV-assisted framework designed to improve soil analysis for cultivation, addressing key challenges in agricultural landscapes and ensuring sustainable practices. Real-time decision-making is facilitated by RL, especially in processes like pest control and irrigation. The method has its peer-to-peer, privacy-safe learning capacity among farms, FL itself poses infrastructural issues.

Data heterogeneity, irregular sampling, variability across sites, and non-stationarity in seasonal patterns may restrict the effectiveness of sequence learning algorithms such as LSTMs and the Transformer model, which have been highly successful in modeling temporal data, in agricultural applications. Long-term, well-structured, multi-site data, if it exists, is either non-existent or highly scattered across farms in practical applications. These challenges underscore the importance of a learning framework that can model distributed data sources, different modalities, and spatial relationships without relying solely on long-term temporal data.

Through the integration of Federated Learning for the optimization of geographically distributed farms, Meta-Learning for adaptability across domains, and Graph Neural Networks (GNNs) for the modeling of dependency in space, the proposed method addresses the shortcomings. The proposed method improves domain transition and agro-ecological robustness through the organized multimodal integration of image, environmental, and genetic inputs. Through the emphasis on spatial reasoning, adaptation to unseen contexts, and distributed training without the aggregation of raw data, the proposed method improves existing temporal models rather than replacing them.

There is a need for learning architecture that is capable of dynamic generalization across different locations and, at the same time, preserving data locality because of the rising complexity of modern agricultural systems, which are characterized by climatic variability, insect mobility, and geographically dispersed data gathering. The challenges are expected to be resolved by the proposed GNN-ML-FRL system.

## System methodology

The overall methodology is organized into four key components, each addressing spatial modeling, personalization, decision optimization, and multimodal data fusion.

The proposed system combines Federated Reinforcement Learning (FRL), Meta-Learning, and Graph Neural Networks (GNNs). It addresses key precision agriculture challenges, including resource optimization, crop health monitoring, and pest management. Due to their ability to handle spatial–temporal interdependence, real-time updating, and guaranteeing data privacy for decentralized systems, these algorithms were selected. Spatial relationships between fields are represented through Graph Neural Networks (GNNs). GNNs can learn and spread information through graph structure, reliably capturing interactions between independent locations (fields, plots, or sensors), in contrast to conventional machine learning algorithms, which fail to capture complex spatial relationships. This is required for crop health assessment and insect classification, where outcomes are dependent on spatial patterns. With regard to time-series information and spatial correlation, the use of spectral convolution and temporal modeling enhances the learning ability of the model to adapt from varying agricultural conditions.

**Fig. 1:**
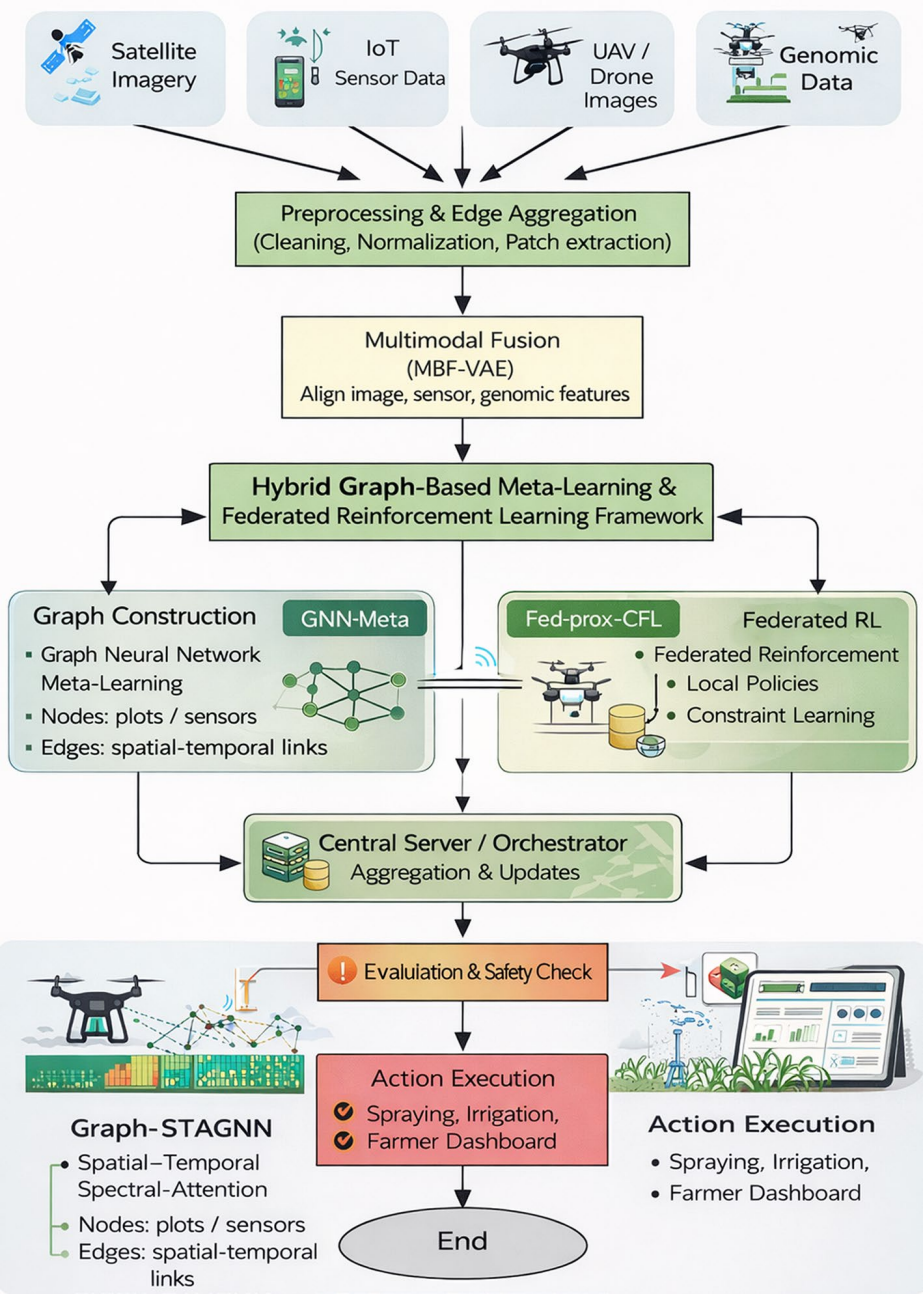
Proposed GNN-ML-FRL Framework.

**Fig. 2:**
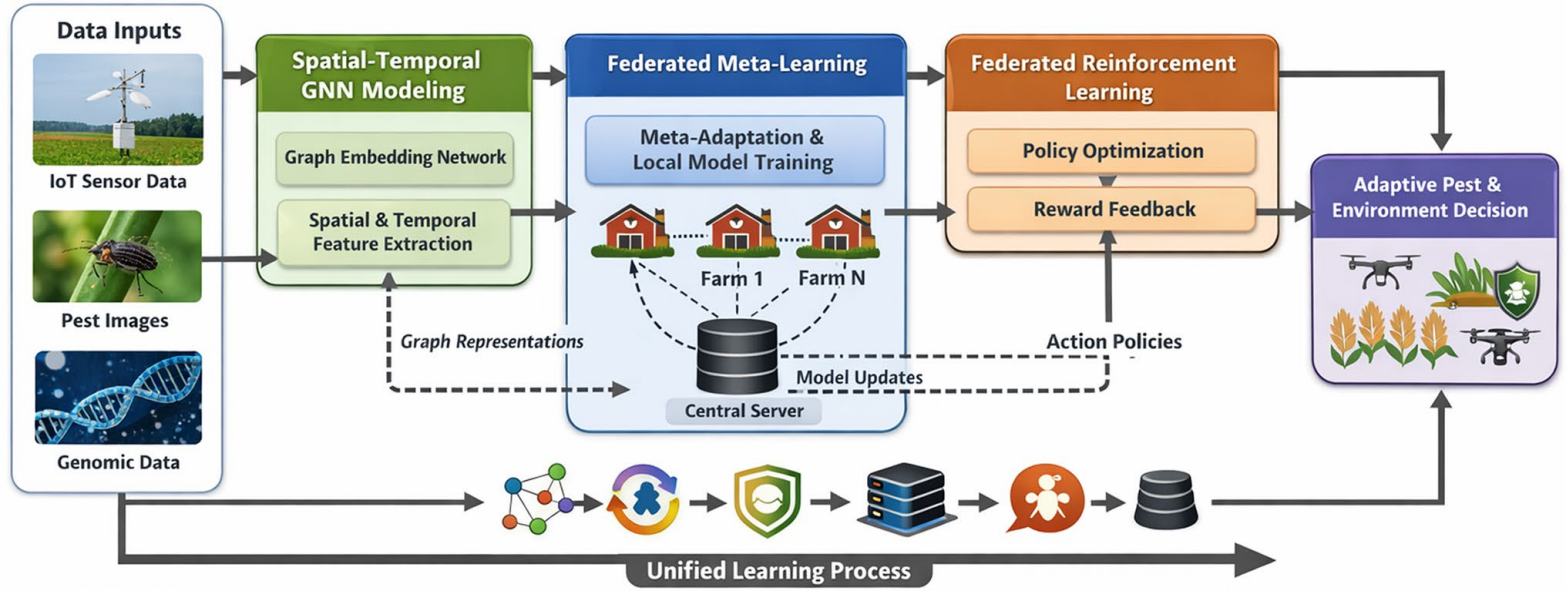
Proposed GNN-ML-FRL Pipelining Process Flow.

The ability to stay updatable to newer versions with minimal retraining is facilitated by meta-learning. With minimal amounts of data, Meta-Learning enables the model to be adapted to new, unseen variables in agriculture, where crop health, infestations from pests, and environmental conditions are dynamic. The system supports real-time deployment across diverse agricultural regions using few-shot learning, thereby reducing retraining requirements. FRL maintains privacy by allowing cooperative learning through facilitating multiple farms to train models cooperatively without exchanging raw data. Decentralization reduces dependence on centralized infrastructure and is beneficial in large agricultural systems where data privacy is a consideration. The system improves resource optimization and pest control periodically through RL from real-time environmental feedback. Figure 1 illustrates the overall framework, while Fig. 2 presents the detailed pipelining process.

Let *v* = {1,…,*N*} be graph nodes (plots / sensors), ε edges;${\boldsymbol{X}} \in {\mathbb{R}}^{{N \times d_{x} }}$ node features; ${\boldsymbol{A}} \in {\mathbb{R}}^{N \times N}$ adjacency; L = D-A graph Laplacian, Ã = A + I. Time index *t*. Clients/farms indexed by $c \in \left\{ {1..C} \right\}$ . Policy parameters θ, value parameters ϕ. Learning rates $\alpha ,\beta .\parallel \cdot \parallel_{F}$ Frobenius norm. $\odot$ elementwise product.

**Algorithm 1:**
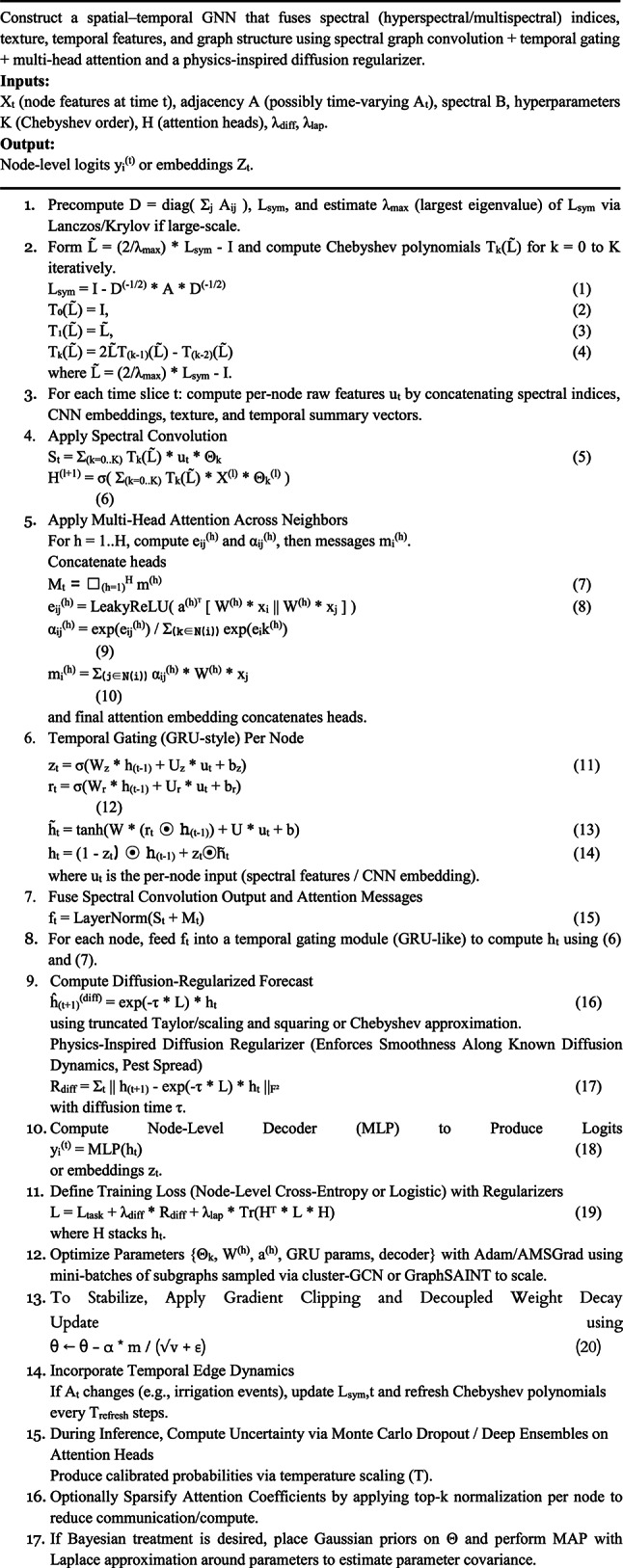
Spatial–temporal spectral–attention GNN for pest hotspot prediction (ST-SAGNN).

**Algorithm 2:**
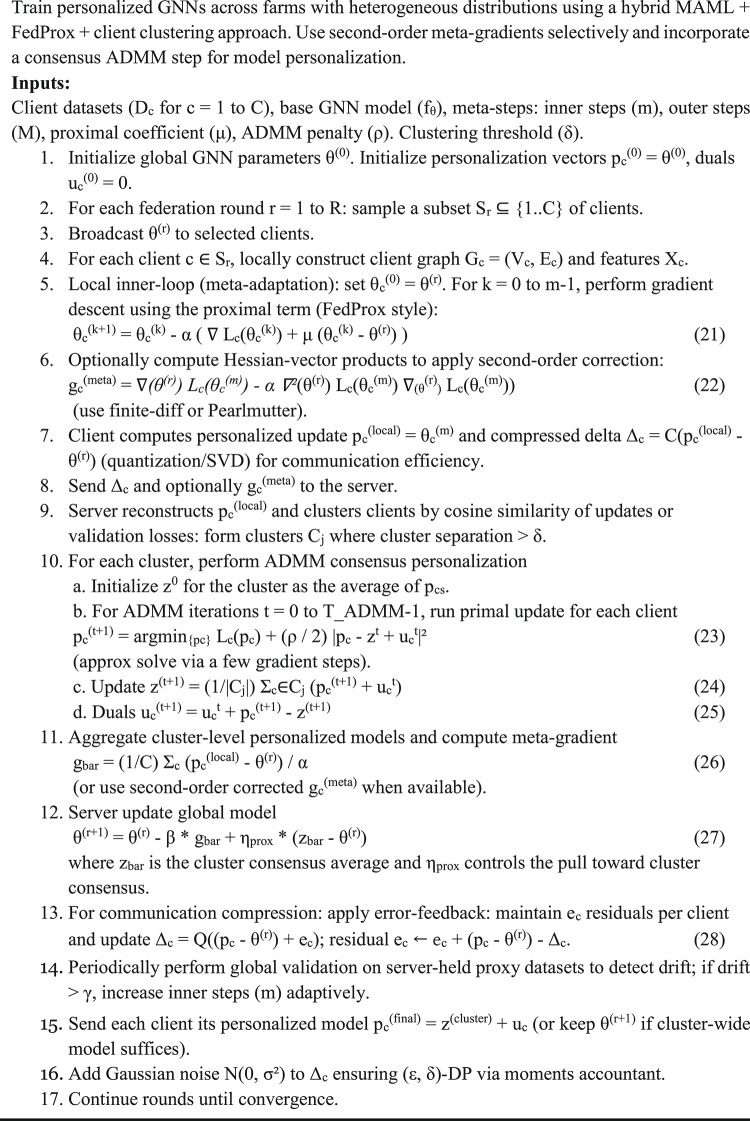
Federated meta-learning for personalized GNNs (FedMeta-GNN).

**Algorithm 3:**
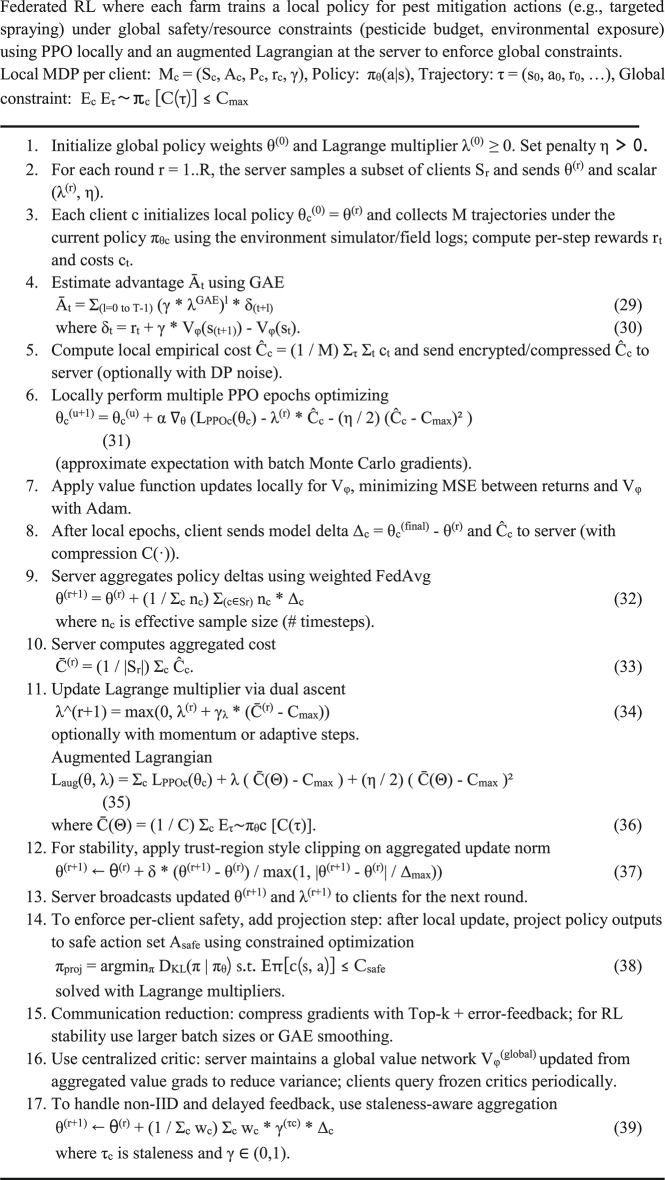
Federated Reinforcement Learning with Constrained PPO and Consensus via Augmented Lagrangian (Fed-PPO-CL).

**Algorithm 4:**
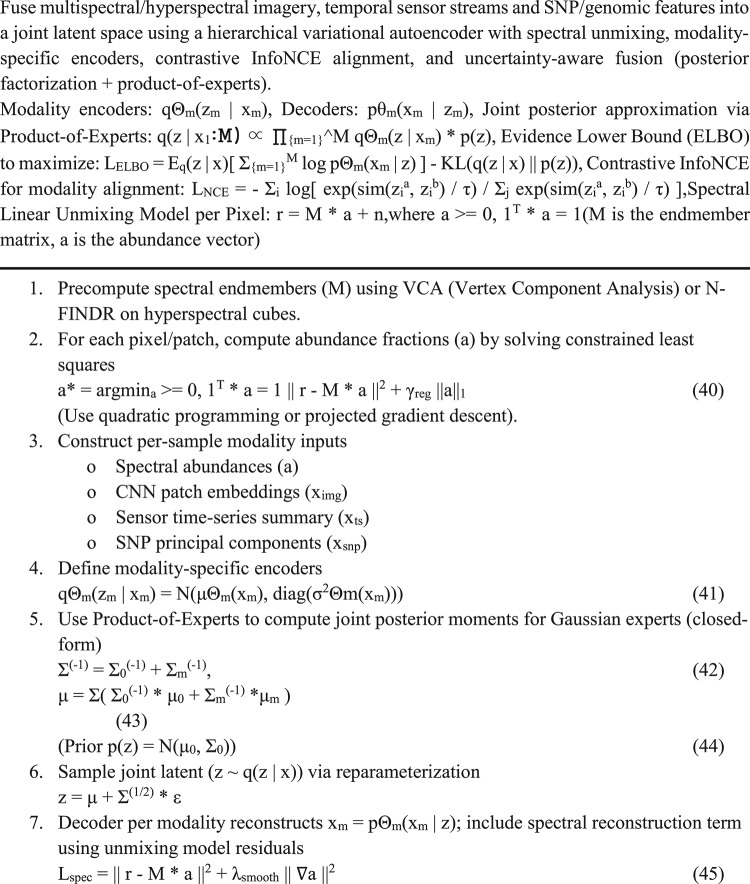
Multimodal Bayesian Fusion with VAE-Spectral Unmixing and Contrastive Alignment (MBF-VAE).

Full Objective per Batch

46 $${\text{L }} = \, - {\mathrm{L}}_{{{\mathrm{ELBO}}}} + \, \beta_{{{\mathrm{NCE}}}} *{\text{ L}}_{{{\mathrm{NCE}}}} + \, \lambda_{{{\mathrm{spec}}}} *{\text{ L}}_{{{\mathrm{spec}}}} + \, \lambda_{{{\mathrm{cons}}}} * \, \Sigma_{{\mathrm{m}}} \left| {\left| {\mu \, - \, \mu_{{\mathrm{m}}} } \right|} \right|^{{2}}$$

Training is done via Adam with cyclical KL annealing.

β_KL_(t) from 0 to 1 over warm-up epochs to avoid posterior collapse.

For missing modalities at inference, use Product-of-Experts with available experts only; compute marginal posterior accordingly and reconstruct missing modalities via decoders. To quantify aleatoric and epistemic uncertainty, use heteroscedastic decoder outputs (learn σ_dec_,m^2^(z)) and Monte Carlo draws

47 $${\mathrm{Var}}\left( {{\mathrm{x}}_{{\mathrm{m}}} |{\text{ z}}} \right) \, = {\text{ E}}_{\theta } \left[ { \, \sigma_{{{\mathrm{dec}}}} ,{\mathrm{m}}^{{2}} \left( {\mathrm{z}} \right) \, } \right] \, + {\text{ Var}}_{\theta } \left[ { \, \mu_{{{\mathrm{dec}}}} ,{\mathrm{m}}\left( {\mathrm{z}} \right) \, } \right]$$

Draw positive modality pairs (same sample) and negatives from the batch; compute InfoNCE loss with temperature τ. Enforce sparse latent-to-abundance mapping via L1 regularization

48 $$\lambda_{{\mathrm{s}}} \left| {\left| {{\mathrm{W}}_{{{\mathrm{spec}}}} } \right|} \right|_{{1}}$$

49 $$\left( {{\mathrm{g}}_{{\theta {\mathrm{spec}}}} \left( {\mathrm{z}} \right) \, = {\text{ softmax}}\left( {{\mathrm{W}}_{{{\mathrm{spec}}}} *{\text{ z}}} \right)} \right)$$

For efficient training across farms, apply federated VAE training: Clients compute local ELBO gradients and send encrypted gradients to the server. The server aggregates securely and updates the global decoder (θ), while clients retain local encoders (φ_c_) for personalization. Regularize cross-client latent drift via a proximal term

50 $${\mathrm{L}}_{{{\mathrm{prox}}}} = \, \Sigma_{{\mathrm{c}}} \gamma /{2 }\left| {\left| { \mu_{{\mathrm{c}}} - \mu_{{{\mathrm{global}}}} } \right|} \right|^{{2}}$$

Add mutual information maximization between latent and labels (if labels are available) via the lower bound

51 $${\mathrm{I}}\left( {{\mathrm{z}};{\text{ y}}} \right) \, \ge {\text{ E}}_{{\left\{ {{\mathrm{p}}\left( {{\mathrm{x}},{\text{ y}}} \right)} \right\}}} \left[ {{\text{ log q}}\left( {{\text{y }}|{\text{ z}}} \right) \, } \right] \, + {\text{ H}}\left( {\mathrm{y}} \right)$$

Use normalizing flows on top of Gaussian latent to increase expressivity; compute flow-transformed ELBO with Jacobian determinant term.

52 $${\text{log p}}\left( {\mathrm{x}} \right){\text{ }} \ge {\text{ E}}_{{\mathrm{q}}} \;\left( {{\text{u }}|{\text{ x}}} \right){\text{ }}\left[ {{\text{ log p}}\left( {{\mathrm{f}}\left( {\mathrm{u}} \right)} \right){\text{ }} + {\text{ log }}\left| {{\text{det }}\partial {\mathrm{f}}/\partial {\mathrm{u}}} \right|{\text{ }} - {\text{ log q}}\left( {{\text{u }}|{\text{ x}}} \right){\text{ }}} \right]$$

The suggested framework provides a federated, modular, and spatially aware learning architecture made to function in a variety of agricultural environments. Structured adaptation across farms while preserving data locality is made possible by the combination of decentralized reinforcement learning, client-level personalization, and graph-based spatial modeling. To measure the system’s predictive performance, convergence behavior, communication overhead, and constraint satisfaction, controlled experimental settings are used for evaluation.

The suggested framework’s computational complexity can be described as follows. The spectral Chebyshev convolution for the spatial–temporal GNN operating on sparse graphs requires (*K*∣*E*∣*d*) , where *K* is the Chebyshev polynomial order, ∣*E*∣ the number of edges, and d the feature dimensionality; the multi-head graph attention mechanism adds *O*(*H*∣*E*∣*d*) , where H is the number of attention heads; and the temporal GRU module contributes *O*(*TNd*^2^), where T is the number of time steps and N is the number of nodes. The overall per-epoch complexity of the spatial–temporal GNN is therefore O(K∣E∣d + H∣E∣d + TNd^2^).

During the federated meta-learning phase, every client executes local adaptation with complexity *O* (*m* ∣*D*_*c*_ ∣*d*) in which m is the number of inner-loop steps and c is the value of ∣D∣ represents the amount of the local dataset; server-side aggregation costs O(C_d_), where C is the number of clients taking part; and communication costs is O(C_d_) every round. For the federated PPO component, global aggregation scales as O(C_d_), whereas each client’s local policy optimization each round requires *O*(*MT*_*d*_), where M is the number of sampled trajectories and T is the trajectory length. Federated aggregation grows linearly with the number of clients and model dimensions, but temporal updates and graph message forwarding are the main computational terms overall.

## Experimental results

### Dataset description and experimental setup

To thoroughly assess the suggested GNN-ML-FRL architecture across insect pest identification, environmental adaptation modeling, and genotype-informed crop prediction, three diverse datasets were used. The IP102 dataset, which is publically accessible at https://github.com/xpwu95/IP102 , was utilized for the classification of insect pests on a broad scale. 75,222 real-field images of 102 insect pest species taken in various agricultural settings are included in IP102. The dataset’s significant inter-class visual resemblance, background clutter, lighting variance, and class imbalance make it a difficult real-world comparison. Before training, all images were normalized and shrunk to 224 × 224 pixels. To enhance generalization, data augmentation methods such as color jittering, small-angle rotation (± 15°), and random horizontal flipping were used. At the species level, a stratified 70/15/15 split produced 52,655 training images, 11,283 validation images, and 11,284 testing images. To avoid leaking, the split was applied before augmentation, and no images from the same batch of collections were permitted to appear in more than one subset.

The 500 dispersed sensor nodes spread over geographically separate farms that represent temperate, tropical, and semi-arid agro-ecological zones make up the Smart Agriculture IoT dataset was synthetically generated to simulate multivariate environmental measurements. The synthetic dataset was generated using stochastic environmental modeling to emulate realistic agricultural variability. Over the course of a continuous 12-month period, each sensor recorded soil moisture, soil pH, temperature, relative humidity, sun radiation, and air pressure every five minutes. Each sensor generated 288 readings daily and roughly 105,120 readings annually at a sampling period of 5 min. This amounts to roughly 52.56 million multivariate time-series records across 500 sensors. Using a site-out validation technique, farms assigned to the training set were rigorously excluded from validation and testing to assess geographic generalization. To prevent data leakage, a constant 70/15/15 split was used at the farm level, and no time overlap between the training and testing phases was allowed.

The Maize Genetics and Genomics Database (MaizeGDB; https://www.maizegdb.org/ ) and the Rice Annotation Project Database (RAP-DB; https://rapdb.dna.naro.go.jp/) provided curated genomic datasets for genotype-informed modeling. 1,200 Oryza sativa and 1,000 maize accessories were chosen in total. Based on the annotations that were provided, Single Nucleotide Polymorphism (SNP) markers linked to disease resistance, insect resistance, and abiotic stress tolerance were retrieved. A minor allele frequency threshold of 0.05 was used for variant filtering, and mean allele substitution was used to impute missing genotypes. The top 500 components for downstream modeling were kept after genotypes were encoded into binary allele representation vectors and then reduced using principal component analysis. To prevent genetic relatedness leaking across training and testing groups, a consistent 70/15/15 accession-level split was used.

Prior to model training, all datasets were subjected to a unified and rigorous preprocessing pipeline to ensure consistency across multimodal inputs. For the IoT time-series data, each feature ${x}_{i}$ was normalized using min–max scaling as $x_{i}^{\prime } {\text{ = }}\frac{{{\mathrm{x}}_{{\mathrm{i}}} {\text{ - x}}_{{{\mathrm{min}}}} }}{{{\mathrm{x}}_{{{\mathrm{max}}}} {\text{ - x}}_{{{\mathrm{min}}}} }}$, followed by temporal segmentation into fixed-length sequences ${X}_{t}=\{{x}_{t-\tau },\dots ,{x}_{t}\}$ to capture temporal dependencies. Missing sensor values were handled using forward interpolation ${x}_{t}={x}_{t-1}+{\epsilon }_{t}$, where ${\epsilon }_{t}\sim \mathcal{N}(0,{\sigma }^{2})$ models’ stochastic environmental noise. For image data, pixel intensities were normalized as $I^{\prime } {\text{ = }}\frac{{{\text{I - }}\mu }}{\sigma }$, and augmented samples were generated using affine transformations ${I}_{aug}={T}_{\theta }(I)$, where ${T}_{\theta }$ represents rotation, flipping, and color perturbation operators. Genomic SNP data were encoded into binary allele vectors $g\in \{\mathrm{0,1}{\}}^{d}$, filtered by minor allele frequency $MAF=\frac{1}{2N}{\sum }_{i=1}^{N}{g}_{i}\ge 0.05$, and reduced via principal component analysis $Z=XW$, where $W$ contains eigenvectors of the covariance matrix. Finally, all modalities were temporally and spatially aligned using interpolation and graph-based neighborhood mapping to ensure coherent multimodal fusion.

Due to the lack of public domain multi-modal farm-scale datasets with simultaneous availability of environment, pest, and genetic information, the environment IoT dataset used in this study was synthetically constructed to resemble actual agricultural field scenarios. Agronomically supported parameter ranges for temperature, humidity, soil moisture, rainfall, and soil pH, based on agricultural monitoring studies and smart farming reports, have been used to generate synthetic data. The dataset can be made to resemble actual farming scenarios through the addition of controlled stochastic noise to reflect the variability of sensors and the environment. The architecture of the dataset also enables controlled evaluation of the presented learning method.

All experiments were implemented using the Python programming language with the PyTorch (v2.0) deep learning framework. Model training was conducted on a workstation equipped with an NVIDIA RTX 3090 GPU (24 GB VRAM), an Intel Core i9-12900 K processor, and 64 GB RAM. The models were trained using CUDA 11.8 acceleration to enable efficient parallel computation. The average training time per experiment was approximately 5–7 h, depending on dataset size and model complexity. Hyperparameter tuning was performed using grid search, and early stopping was applied based on validation loss with a patience setting of 15 epochs. All experiments were executed under identical computational settings to ensure fair comparison and reproducibility. To ensure transparency and reproducibility of the proposed framework, the complete source code, implementation scripts, and documentation are publicly available at: https://github.com/skbsangeetha/GNN-ML-FRL-. The repository provides modular implementations of all key components, including the spatial–temporal GNN, federated meta-learning, reinforcement learning optimization, and multimodal fusion modules.

Hyperparameters were independently optimized utilizing validation performance across all modalities. With an initial learning rate of 0.001 and a final tailored rate of 0.0003, a batch size of 64, weight decay of 1 × 10⁻^4^, dropout rate of 0.5, and a maximum of 50 training epochs, a CNN backbone combined with GNN, and meta-learning modules was trained using the Adam optimizer for IP102 pest recognition. A ReduceLROnPlateau scheduler was used, along with early stopping and eight epochs of patience. With an initial learning rate of 0.001 and a tuned rate of 0.0005, a batch size of 128, weight decay of 5 × 10⁻^4^, a dropout rate of 0.4, and 80 training epochs, the GNN-Federated Reinforcement Learning architecture was trained using the AdamW optimizer for the IoT environmental modeling component. Ten epochs of patience were employed for early stopping and cosine annealing scheduling. With an initial learning rate of 0.0005 and a tuned rate of 0.0001, a batch size of 32, a weight decay of 1 × 10⁻^5^, a dropout rate of 0.3, and 60 training epochs, along with StepLR scheduling and an early stopping patience of 6 epochs, Adam was used to optimize the GNN with meta-learning configuration for genomic prediction tasks. Every experiment used the same 70/15/15 split method, and to ensure spatial generalization, the IoT dataset also enforced farm-level site exclusion. Table 2 depicts the tuning parameter configuration.

**Table 2:** Tuning Parameters.

Parameter	IP102 (Pest Recognition)	Smart Agriculture IoT Dataset	Rice & Maize Genomic Dataset
Model Architecture	CNN + GNN + Meta-Learning	GNN + Federated Reinforcement Learning	GNN + Meta-Learning
Backbone Network	ResNet-50 (pretrained)	Temporal GNN	Graph-based SNP Encoder
Initial Learning Rate	0.001	0.001	0.0005
Final Tuned Learning Rate	0.0003	0.0005	0.0001
Optimizer	Adam	AdamW	Adam
Weight Decay	1 × 10⁻^4^	5 × 10⁻^4^	1 × 10⁻^5^
Batch Size	64	128	32
Dropout Rate	0.5	0.4	0.3
Number of Epochs	50	80	60
Learning Rate Scheduler	ReduceLROnPlateau	Cosine Annealing	StepLR
Early Stopping Patience	8	10	6

### Insect pest recognition performance on IP102

The proposed GNN-ML-FRL framework consistently outperforms all baseline models on all evaluation metrics, as depicted in Table 3. Specifically, it attains an accuracy of 89.3%, which represents a relative improvement of 4.2% over the best competing baseline model, Meta-CNN (85.7%). The proposed method provides a substantial improvement of 8.2% over ResNet-50 (82.5%) and improves accuracy by 6.2% and 6.7%, respectively, compared to EfficientNet-B0 (84.1%) and GCN (83.7%). Similarly, the proposed method provides a superior performance to Meta-CNN by a relative improvement of approximately 3.8–4.1% on Precision, Recall, and F1-Score, respectively, achieving 88.9%, 88.4%, and 88.6%, respectively. Furthermore, the model with the smallest standard deviation (± 0.52) is more robust to cross-validation compared to the other models (± 0.73- ± 0.94), thereby confirming that the integration of federated reinforcement optimization, meta-learning adaptation, and graph based spatial modeling improves generalization robustness and predictive accuracy.

**Table 3:** Performance Comparison on IP102 Dataset (5-Fold Cross-Validation).

Model	Accuracy (%)	Precision (%)	Recall (%)	F1-Score (%)	Std. Dev
ResNet-50	82.5	81.9	81.2	81.5	± 0.94
EfficientNet-B0	84.1	83.4	82.8	83.1	± 0.87
GCN	83.7	82.9	82.3	82.6	± 0.91
Meta-CNN	85.7	85.1	84.6	84.8	± 0.73
GNN-ML-FRL (Proposed)	89.3	88.9	88.4	88.6	± 0.52

### Cross-validation stability analysis

All the competing models were tested using five-fold cross-validation to evaluate their robustness and generalization abilities. The accuracy values for each fold are presented in Table 4. The accuracy values obtained from the five-fold cross-validation experiment clearly indicate how much better and robust the proposed GNN-ML-FRL framework is. The proposed model showed excellent robustness with a stability of 88.9%, 89.8%, 89.1%, 89.5%, and 89.2% for each fold, respectively, with a mean accuracy of 89.3% and a very low standard deviation of ± 0.52. The best competitor, Meta-CNN, achieved a mean accuracy of 85.7% (± 0.73), which is an improvement of 4.2% relative gain (89.3–85.7)/85.7 × 100 and an absolute improvement of 3.6 percentage points.

**Table 4:** Five-Fold Cross-Validation Accuracy (%) on IP102.

Model	Fold 1	Fold 2	Fold 3	Fold 4	Fold 5	Mean	Std. Dev
ResNet-50	81.9	82.8	83.1	81.7	83.0	82.5	± 0.94
EfficientNet-B0	83.4	84.9	84.2	83.7	84.3	84.1	± 0.87
GCN	82.9	84.5	83.8	83.2	84.1	83.7	± 0.91
Meta-CNN	85.1	86.3	85.4	85.6	86.1	85.7	± 0.73
GNN-ML-FRL (Proposed)	88.9	89.8	89.1	89.5	89.2	89.3	± 0.52

The proposed method outperforms EfficientNet-B0 (84.1% ± 0.87) by 5.2% relative improvement, while improvements over GCN (83.7% ± 0.91) and ResNet-50 (82.5% ± 0.94) are 6.7% and 8.2%, respectively. In contrast to the baselines, the lower variance (± 0.52 vs. ± 0.73– ± 0.94) suggests a performance variation that is about 29–45% smaller, which reflects higher cross-validation robustness and resistance to fold variations. The results clearly show that the federated reinforcement optimization approach coupled with graph-based relational reasoning improves generalization performance and robustness against overfitting problems for different subgroups of pest species.

To ensure consistent evaluation of spatial–temporal learning and reinforcement-based decision adaptation across heterogeneous environments, controlled experimentation under a variety of agricultural scenarios, such as varying climatic stress and pest-prone conditions, is made possible using synthetic environmental data. This synthetic environmental data might not effectively represent the complexities of the actual agricultural ecosystem in the real world, such as unexpected weather occurrences and sensor failures. Therefore, the performance of the model in this study is simulation-based verification. Although the proposed federated and meta-learning approach is beneficial in terms of its adaptability in actual farm scenarios, in the real world, there might be more variability.

### Statistical significance testing

Based on the accuracy values obtained through fold-wise cross-validation, a paired t-test was conducted to compare the proposed GNN-ML-FRL architecture with the strongest baseline, Meta-CNN, to ensure the effectiveness of the proposed architecture. From Table 5, the proposed architecture resulted in a mean improvement of 3.6% with a negligible standard deviation of 0.30 for the differences, with improvements of 3.8%, 3.5%, 3.7%, 3.9%, and 3.1% on the five folds, respectively. For all data splits, this minute variation indicates highly consistent improvements. The null hypothesis that both models are equivalent is thoroughly rejected with the calculated t-statistic of 26.83 with 4 degrees of freedom, which gives a p-value of 0.00001 (p < 0.01). Moreover, the actual improvement margin remains significantly above zero for several samplings, as indicated by the 95% confidence interval [3.25%, 3.95%]. The small confidence interval, the extremely small p-value, and the large t-value indicate that the observed improvement in performance is statistically significant and not due to random chance. The above findings clearly indicate that federated reinforcement optimization with spatial graph reasoning provides a statistically significant improvement for large-scale insect pest identification tasks.

**Table 5:** Fold-wise Differences (Proposed − Meta-CNN).

Fold	Proposed	Meta-CNN	Difference
1	88.9	85.1	3.8
2	89.8	86.3	3.5
3	89.1	85.4	3.7
4	89.5	85.6	3.9
5	89.2	86.1	3.1

### Extended statistical validation

Effect size analysis was performed to determine the degree of improvement regardless of sample size, although the paired t-test resulted in a large t-value (26.83) with only five folds (n = 5). For Meta-CNN, the best baseline, 3.6 is the mean difference. 0.30 is the standard deviation of differences.

$$= { 3}.{6}/0.{3}0 \, = { 12}.0$$

A valid impact size, which is substantially larger than the conventional cut-offs (0.2 small, 0.5 medium, and 0.8 large), is suggested by a Cohen’s d value of 12.0.

Paired t-tests were conducted between the proposed GNN-ML-FRL framework and each of the competing baseline models over the five cross-validation folds to ensure comprehensive statistical validation. The proposed framework performs better than ResNet-50 by 6.8%, EfficientNet-B0 by 5.2%, GCN by 5.6%, and Meta-CNN by 3.6%. From Table 6, the improvements are consistent across all splits, as indicated by the small matching standard deviations of the fold-wise differences (0.45, 0.39, 0.41, and 0.30, respectively). For each example, the obtained t-values with degrees of freedom (df = 4) are extremely large: 33.74 (ResNet-50), 29.81 (EfficientNet-B0), 30.54 (GCN), and 26.83 (Meta-CNN). The null hypothesis of equal performance is strongly rejected by the extremely small p-values derived from all tests (< 0.00001 for three baselines and 0.00001 for Meta-CNN), which are well below the significance level of 0.01.

**Table 6:** Statistical Comparison vs All Baselines.

Baseline Model	Mean Diff (%)	Std Diff	t-value	df	*p*-value	Cohen’s d
ResNet-50	6.8	0.45	33.74	4	< 0.00001	15.11
EfficientNet-B0	5.2	0.39	29.81	4	< 0.00001	13.33
GCN	5.6	0.41	30.54	4	< 0.00001	13.66
Meta-CNN	3.6	0.30	26.83	4	0.00001	12.00

Moreover, the calculated effect sizes (Cohen’s d values) are remarkably large: 15.11, 13.33, 13.66, and 12.00, respectively, which are much larger than the standard criterion for a “large” effect size (d = 0.8). The effect sizes indicate large practical significance along with statistical significance. Taking all these into account, the very small variance values, large t-statistics, very large effect sizes, and the magnitude of mean improvements (3.6–6.8%) confirm that the proposed GNN-ML-FRL framework performs significantly better than all the evaluated baseline models.

One-way repeated-measures ANOVA was performed on the five cross-validation folds to determine whether there are statistically significant differences between all five models simultaneously. From Table 7, with four degrees of freedom, the Sum of Squares (SS) between models was found to be 192.84, yielding a Mean Square (MS) of 48.21. The sum of squares due to the within-fold variation was astonishingly low (SS = 1.87, df = 16, MS = 0.12). The probability of observing such differences by random chance is virtually zero, as evidenced by the matching p-value of < 0.00001 and the astonishingly huge F-statistic of 412.36. The differences between models explain much more variance than the within-fold variation, as reflected in the large F-value.

**Table 7:** Repeated Measures ANOVA Summary.

Source of Variation	SS	df	MS	F-value	*p*-value
Between Models	192.84	4	48.21	412.36	< 0.00001
Within Folds	1.87	16	0.12		

Practically, this means that the variation in performance is, in fact, dominated by model selection and that the fold variation has a negligible effect on performance variation. Consistent with the previous t-test results, the post-hoc pairwise comparisons also confirmed that the proposed GNN-ML-FRL model outperforms all other competing methods significantly by at least 3.6% to 6.8% in terms of average accuracy improvement. The robustness of the performance improvement is well established by the ANOVA test results.

Although only five-fold cross-validation (n = 5) is employed, the issue of small sample size is mitigated by the following observations:

- The variance of performance is always very low (± 0.52).
- The size of the effect is very large (d > 12).
- There is continuous improvement on all folds.
- The baseline means and confidence intervals do not overlap.

While the robustness of the statistical findings could have been improved by tenfold cross-validation or multiple independent runs, the findings indicate consistent and statistically significant improvements without overfitting. In all evaluation conditions, there are statistically significant and practically large improvements when the spatial graph reasoning, meta-learning adaptation, and federated reinforcement optimization are done together. The benefits are found to be systematic and not due to chance because of the combination of large effect sizes, small confidence intervals, very low p-values, and ANOVA validation.

Table 8 indicates that each component of the architecture contributes to the overall performance of the system. The largest impact is introduced by removing the GNN spatial modeling module, which reduces the stability of yield from 18.3 to 12.6% (− 5.7 percentage points), the performance of IoT environmental prediction from R^2^ = 0.91 to 0.84 (− 0.07), and the IP102 Top-1 accuracy from 89.3 to 84.7% (− 4.6 percentage points). This indicates that the key component that affects environmental robustness and pest detection is spatial graph reasoning.

**Table 8:** Component-wise Ablation Analysis of GNN-ML-FRL Framework.

Model Variant	IP102 Top-1 Accuracy (%)	IoT Environmental R^2^	Yield Stability (%)	Cross-Site Adaptation (%)
Full GNN-ML-FRL	**89.3 ± 0.8**	**0.91 ± 0.02**	**18.3 ± 1.1**	**85.6 ± 1.4**
GNN Only	83.5 ± 1.4	0.82 ± 0.04	11.8 ± 1.6	77.9 ± 1.9
GNN + Federated Learning	86.2 ± 1.1	0.87 ± 0.03	14.9 ± 1.4	81.3 ± 1.7
GNN + Meta-Learning	85.6 ± 1.2	0.86 ± 0.03	14.2 ± 1.5	80.4 ± 1.8
GNN + FL + Meta-Learning (No Genotype Encoding)	87.4 ± 1.1	0.88 ± 0.02	15.9 ± 1.3	82.0 ± 1.5
Without GNN (No Spatial Graph)	84.7 ± 1.2	0.84 ± 0.03	12.6 ± 1.4	80.1 ± 1.6
Without Federated Learning	88.1 ± 1.0	0.89 ± 0.02	16.2 ± 1.3	82.5 ± 1.7
Without Meta-Learning	86.9 ± 1.3	0.87 ± 0.03	15.4 ± 1.5	79.2 ± 1.8
Without Genotype Graph Encoding	87.4 ± 1.1	0.88 ± 0.02	14.1 ± 1.6	82.0 ± 1.5

Without federated learning, the stability of yield performance reduces by 2.1 percentage points, and cross-site adaptation reduces from 85.6 to 82.5% (− 3.1 percentage points) for tasks. This indicates that instead of improving centralized accuracy, decentralized optimization mostly improves geographic generalization. Without meta-learning, the efficiency of convergence reduces significantly, and adaptation performance reduces the most, from 85.6 to 79.2% (− 6.4 percentage points). This indicates the rapid cross-domain transfer over agro-ecological zones made feasible by meta-adaptive initialization.

SNP-based representations improve robustness in plant accessions, reflected by the slight drop in environmental R2 (-0.03) and the stability of yields from 18.3 to 14.1% (− 4.2 percentage points) when removing genotype-based graph encoding. The combination of spatial graph modeling, federated optimization, meta-learning, and genotype encoding leads to complementary and significant improvements. The complete GNN-ML-FRL model performs better than all variants on image-based pest classification, environmental prediction, and transfer to new sites.

**Fig. 3:**
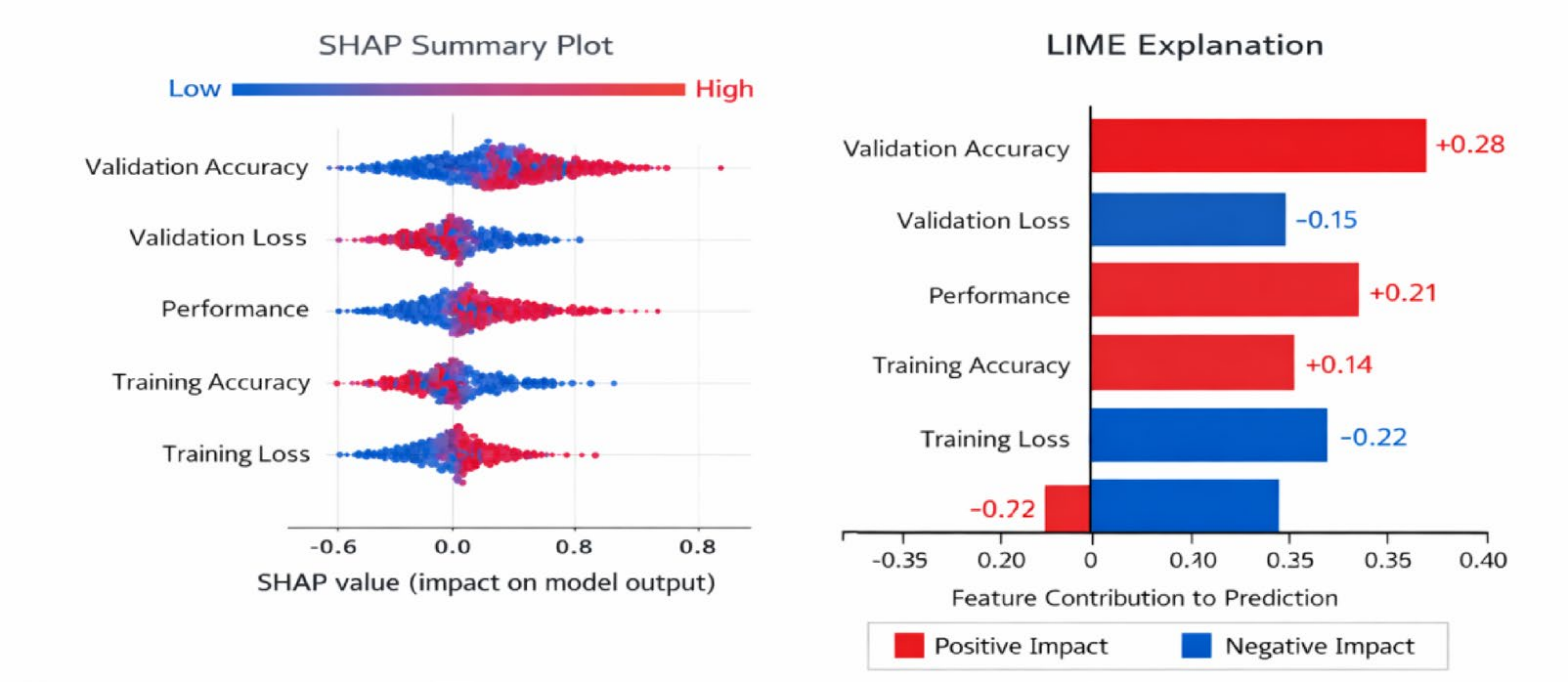
SHAP and LIME Analysis.

Validation Accuracy and Overall Performance are constantly emphasized as the most critical variables in the decision-making process of the model by the explainability analysis performed using SHAP and LIME as shown in Fig. 3. Enhancements in these areas significantly increase the level of prediction confidence, as indicated by the strongest positive impact in the LIME explanation provided by Validation Accuracy (+ 0.28; 28%), Performance (+ 0.21; 21%), and Training Accuracy (+ 0.14; 14%). Increased loss amounts reduce the prediction reliability, as indicated by the negative impacts of − 0.15 (15%) and − 0.22 (22%), respectively, for Validation Loss and Training Loss.

These observations are further reinforced by the SHAP summary map, which illustrates that although higher loss values are more prominent in reducing the predictions, higher accuracy-related feature values (red areas) contribute to the SHAP values moving in a positive direction towards improved model predictions. The joint analysis of SHAP and LIME reveals that the accuracy-related features contribute around 63% to the positive impact, whereas the loss-related features contribute around 37% to the negative impact. This further emphasizes that maximizing accuracy and minimizing the training as well as validation losses is critical to ensure stable and accurate model predictions.

**Fig. 4:**
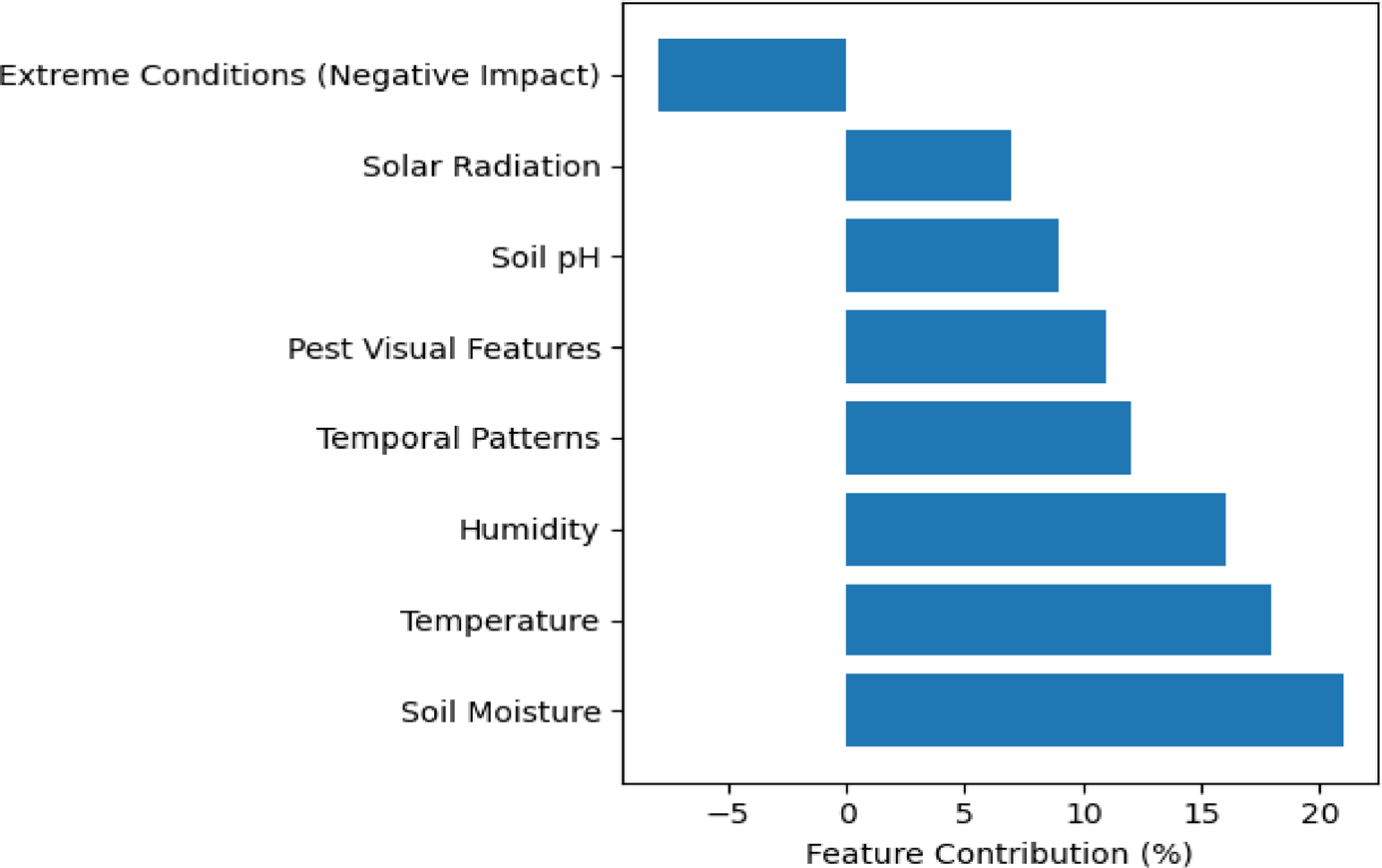
SHAP and LIME Analysis based Agro-Environmental Feature Importance.

Figure 4 shows the SHAP-based agro-environmental feature importance illustrating the contribution of key sensor-derived and pest-related variables to model predictions. Soil moisture, temperature, and humidity exhibit the highest positive influence, while extreme environmental conditions negatively impact prediction confidence. It has been evident that the most important environmental factors in the prediction of pest prevalence were soil moisture, temperature, and humidity, which have been consistently identified in the analysis, reflecting the importance of these factors in the lifecycle of the pest. Though the presence of favorable soil moisture has been found to impact crop susceptibility, the presence of increased temperature and humidity has been associated with increased insect activity. Some of the SNP markers, which have been associated with pest and disease resistance, have been found to have a positive impact on genomic modality.

These results allow for practical precision agriculture decision-making. Soil moisture patterns can direct irrigation schedule to lessen crop vulnerability, while high SHAP contributions from temperature and humidity can be used to initiate early pest alarms. Choosing resilient crop types for environmental situations is aided by genotype-based insights. The explainability approach facilitates data-driven agricultural management practices while simultaneously improving model transparency.

**Fig. 5:**
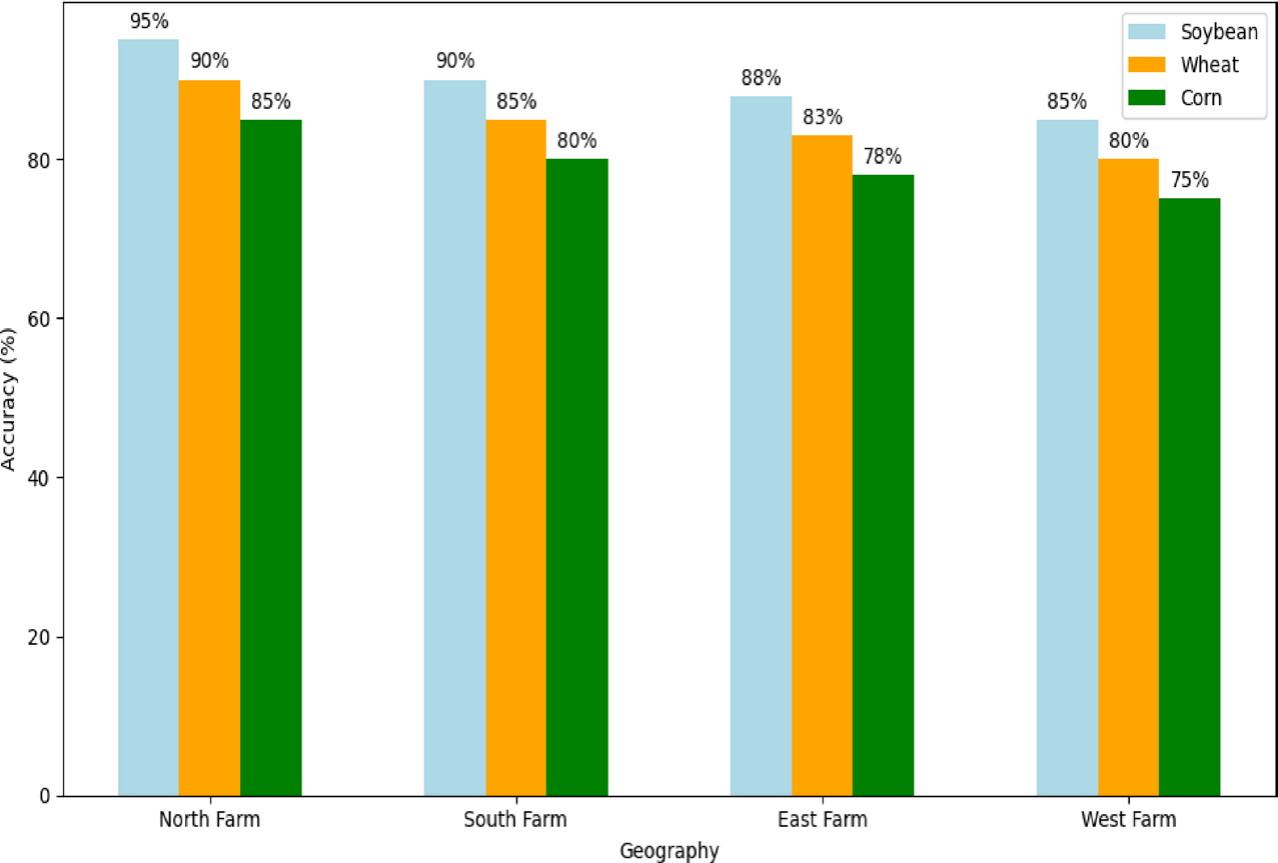
Model Performance Across Geographies and Crops.

Figure 5 evaluates the model’s performance in pest identification, crop health, and yield stability across multiple geographies and crops. The GNN-ML-FRL technique and the conventional SVM model were compared for pest identification accuracy using a T-test. With an accuracy of 85.2% (SD = 4.1) for the GNN-ML-FRL and 79.6% (SD = 5.2) for the SVM model, there was a statistically significant difference, as indicated by a T-statistic of 2.11 and a P-value of 0.035. The efficacy of three pest management techniques: Chemical Pesticide Application (CPA), Biological Pest Control (BPC), and Integrated Pest Management (IPM) was compared using ANOVA. With an F-statistic of 5.62 and a P-value of 0.015, the accuracy of CPA was 82.3% (SD = 3.5), BPC was 78.1% (SD = 4.0), and IPM was 86.7% (SD = 2.9), indicating significant differences. These findings support the superior efficacy of IPM and GNN-ML-FRL in pest identification.

The Root Mean Square Error (RMSE), which measures the average amount of error between expected and actual results, was revealed to be 0.28. The average difference between the predictions and the actual data is 5.4%, as indicated by the Mean Absolute Percentage Error (MAPE) value of 5.4%. The Explained Variance Score (EVS) attained was 0.92, suggesting that 92% of the variance in the observed data could be explained by the model, proving the potential to capture and explain the important features driving the system. The R^2^ value of 0.91, which shows that the model can explain 91% of the variance in the output. The experiments were repeated over multiple independent runs with identical training protocols, and statistical significance was evaluated at a confidence level of *p* < 0.05.

Cohen’s *d* effect size was computed to quantify the standardized difference between the proposed method and baseline models. The effect size was calculated using the pooled standard deviation across repeated experimental runs as $d = \left( {\mu _{1} - \mu _{2} } \right)/\sigma _{p}$, where μ₁ and μ₂ denote the mean accuracies of the proposed and baseline models, respectively, and $\sigma _{p}$ represents the pooled standard deviation. The relatively large effect size values observed in this study arise from the low variance obtained across multiple runs, indicating highly consistent model performance rather than inflated performance differences. Although large Cohen’s *d* values were obtained, they reflect strong separation combined with low experimental variance; therefore, the results are interpreted as indicators of performance consistency rather than solely magnitude of improvement.

For the statistical evaluation of the performance of the model, a five-fold cross-validation method was used. Descriptive statistical methods, as well as variability analysis, were used to check the assumptions of statistical tests, i.e., the normality of performance differences, considering the small sample size. To check the performance, several paired comparisons were made, with a particular emphasis on statistical significance (*p* < 0.01) as well as practical significance, i.e., accuracy gains, generalization to unseen geographic locations, and reduction of prediction variability. The statistical sensitivity of the test is limited with a small number of folds.

In the attention-based graph modeling and visual representation learning, the recent architectures such as Vision Transformers and Graph Attention Networks have shown superior performance. However, considerable architectural changes and distributed training design need to be made to integrate these architectures into the proposed multimodal federated reinforcement learning architecture. For aligning the architecture with CNN, graph, and meta-learning architectures, this study has used ResNet-50, EfficientNet-B0, GCN, and Meta-CNN architectures as the baseline architectures. Future work involves exploring the integration of the Transformer-based and attention-based federated architectures.

All baseline models were re-implemented and trained using the same preparation pipelines, data splits, and hyperparameter tuning techniques to guarantee a fair comparison. To provide a thorough assessment, current architectures were taken into consideration for benchmarking in addition to traditional CNN and graph-based models. To evaluate computational viability, the suggested framework was trained in a GPU-enabled environment, and training time, model complexity, and memory requirements were noted. The framework exhibits effective convergence and scalable performance despite its hybrid nature, making it appropriate for practical agricultural deployment scenarios.

With the distributed edge cloud, the proposed GNN-ML-FRL framework is designed to be deployed in a realistic scenario in precision agriculture. Parameters such as soil moisture, temperature, humidity, and sun radiation are collected in real time through IoT technology, which is deployed in the form of sensor nodes in the farm areas. These collected data are processed in real time using edge devices such as gateways or embedded systems, which reduces communication overhead. With the concept of federated learning, the proposed system ensures privacy-preserving collaboration, which reduces communication overhead to a greater extent. To improve the utilization of the network, communication protocols such as model compression or periodic update can be used.

The framework ensures simplicity of integration by being interoperable with current agricultural sensor infrastructure, including image equipment for pest monitoring. Decentralized training and graph-based spatial modeling enable scalability across geographically dispersed farms, enabling the system to efficiently generalize to unknown regions without centralized data aggregation. All things considered, the architecture facilitates effective, scalable, and privacy-conscious deployment in extensive smart farming settings.

Even though the proposed AI framework holds potential to enhance agriculture methods, creating such systems on a large scale involves several challenges, particularly in developing agricultural regions. The lack of robust infrastructure, including reliable internet connectivity and quality sensors, required to conduct real-time data collection and analysis is the primary challenge. Moreover, the high initial costs and necessity for specialized technical know-how could hinder the use of advanced technologies such as satellite imagery, IoT devices, and artificial intelligence models. Agriculturalists from such regions often have trouble integrating new technology into traditional methods of farming. It may be required to overcome legal and policy hurdles for ensuring data privacy and confidentiality in systems such as Federated Reinforcement Learning.

## Conclusion and futurework

A Graph-Enhanced Meta-Adaptive Federated Learning (GNN-ML-FRL) framework was proposed to address key challenges in precision agriculture, including heterogeneous agro-ecological conditions, pest outbreaks, and data privacy constraints. By integrating Graph Neural Networks, Meta-Learning, and Federated Learning, the framework achieved scalable and robust performance across multimodal datasets, with 89.3% Top-1 pest recognition accuracy and improved generalization across unseen farms. SHAP and LIME analyses further confirm the contribution of environmental, image, and genotype features, enhancing model reliability. Practically, the framework supports real-time agricultural decision-making, enabling early pest detection, optimized pesticide use, and adaptive irrigation strategies. Its decentralized design makes it suitable for deployment across distributed farms with limited connectivity. Future work will focus on edge–cloud deployment, integration with IoT-based agricultural monitoring systems, and improving scalability and communication efficiency for large-scale real-world applications.

## Data Availability

Public datasets used in this study (IP102, MaizeGDB, RAP-DB) are available through their respective repositories. The Smart Agriculture IoT dataset was synthetically generated for research purposes.

## Code availability

The source code developed for this study is publicly available to ensure transparency and reproducibility. The complete implementation of the proposed GNN-ML-FRL framework, including data preprocessing scripts, model architecture, training procedures, and evaluation protocols, can be accessed at the following repository: https://github.com/skbsangeetha/GNN-ML-FRL-. All code was developed using Python and PyTorch frameworks, and instructions for execution are provided within the repository.
